# Rolling Bearing Degradation Identification Method Based on Improved Monopulse Feature Extraction and 1D Dilated Residual Convolutional Neural Network

**DOI:** 10.3390/s25144299

**Published:** 2025-07-10

**Authors:** Chang Liu, Haiyang Wu, Gang Cheng, Hui Zhou, Yusong Pang

**Affiliations:** 1 School of Mechanical and Electrical Engineering, Xuzhou University of Technology, Xuzhou 221116, China; liuchang@xzit.edu.cn; 2 School of Electrical and Mechanical Engineering, China University of Mining and Technology, Xuzhou 221116, China; ts22050183p31@cumt.edu.cn (H.W.);; 3 School of Chemical Engineering and Technology, China University of Mining and Technology, Xuzhou 221116, China; 4 Faculty of Mechanical Engineering, Delft University of Technology, 2628 CD Delft, The Netherlands; y.pang@tudelft.nl

**Keywords:** feature extraction, degradation identification, dilated convolution, residual connection

## Abstract

To address the challenges of extracting rolling bearing degradation information and the insufficient performance of conventional convolutional networks, this paper proposes a rolling bearing degradation state identification method based on the improved monopulse feature extraction and a one-dimensional dilated residual convolutional neural network (1D-DRCNN). First, the fault pulse envelope waveform features are extracted through phase scanning and synchronous averaging, and a two-stage grid search strategy is employed to achieve FCC calibration. Subsequently, a 1D-DRCNN model is constructed to identify rolling bearing degradation states under different working conditions. The experimental study collects the vibration signals of nine degradation states, including the different sizes of inner and outer ring local faults as well as normal conditions, to comparatively analyze the proposed method’s rapid calibration capability and feature extraction quality. Furthermore, t-SNE visualization is utilized to analyze the network response to bearing degradation features. Finally, the degradation state identification performance across different network architectures is compared in pattern recognition experiments. The results show that the proposed improved feature extraction method significantly reduces the iterative calibration computational burden while effectively extracting local fault degradation information and overcoming complex working condition influence. The established 1D-DRCNN model integrates the advantages of dilated convolution and residual connections and can deeply mine sensitive features and accurately identify different bearing degradation states. The overall recognition accuracy can reach 97.33%.

## 1. Introduction

The rolling bearing is one of the critical components in rotating machinery and transmission systems. Its health condition directly affects the operational safety of the entire equipment. The harsh work environment accelerates bearing performance degradation and leads to faults, unplanned downtime, maintenance, or even accidents that may cause significant economic losses and personnel injuries [1]. Therefore, the accurate identification of bearing fault types and degradation states is essential to provide technical support for the development and application of predictive maintenance technologies and has become a key research focus in the electromechanical equipment fault diagnosis field [2].

To extract the bearing fault and degradation state information contained in vibration signals, various feature extraction methods have been proposed, including time-domain [3], frequency-domain [4], time-frequency domain [5], and entropy-based feature indicators [6], as well as various feature selection and multi-feature fusion techniques [7,8,9]. These methods have been widely applied in fault diagnosis, degradation identification, and the remaining useful life prediction. However, many of these approaches focus on signal differences rather than the underlying fault information, making them difficult to adapt to the complex working condition influence, thus significantly limiting their applicability.

Some researchers prefer methods with clear physical significance. Based on the bearing dynamics theory, they have investigated the vibration mechanisms of local bearing faults, the mapping relationship of monopulse detailed features, and related diagnostic techniques, enabling the direct measurement of local fault size from vibration signals [10,11,12]. However, obtaining a high-quality monopulse typically requires extensive manual selection, and the detection results often rely heavily on subjective judgment. Some studies have explored monopulse waveform feature extraction methods, but their practical application effects still need to be verified and improved [13,14].

After feature extraction, high-performance deep learning models have been widely applied to various pattern recognition tasks [15]. Convolutional neural networks (CNNs) are commonly used in image recognition and processing tasks and are also used in the fault diagnosis field due to their many advantages [16,17]. Wang et al. [18] employed a 1D-CNN for multimodal sensor data fusion and diagnosed bearing fault types based on both vibration and acoustic signals. Their method achieved higher accuracy in noisy environments compared to single-modality inputs. Luo et al. [19] combined Variational Mode Decomposition (VMD) and Dual-Channel CNN (DC-CNN) for dual-feature extraction, enhancing the approach’s robustness. Some high-performance network models have been further adopted for bearing degradation identification and remaining useful life (RUL) prediction. Cheng et al. [20] proposed a nonlinear degradation indicator constructed and estimated through CNN and used a Bi-directional Long Short-Term Memory (BiLSTM) network for bearing health prognosis. Zhu et al. [21] introduced a dynamically activated convolutional network (DACN) for feature extraction and integrated it with a ConvLSTM model for accurate RUL prediction. Lv et al. [22] designed a CNN model incorporating both temporal and dimensional attention mechanisms, which effectively exploited multisensor degradation data under varying working conditions for bearing state prediction. However, the computational complexity and total number of parameters in deep learning networks increase rapidly with network depth, and deeper architectures do not necessarily lead to better results [23,24]. Furthermore, due to the existence of multiple degradation states and the high similarity between sample features, how to deeply mine detailed feature information remains a key research challenge.

To address the aforementioned challenges, this paper proposes a rolling bearing degradation state recognition method based on an improved monopulse waveform feature extraction and one-dimensional dilated residual convolutional neural network (1D-DRCNN). First, a two-stage grid search strategy is employed to accelerate the extraction of monopulse envelope waveform features. Then, the 1D-DRCNN is utilized to identify the degradation states of rolling bearings. The proposed method is able to overcome the influence of varying rotational speeds, loads, and variable-speed operating conditions. It can accurately identify bearing degradation states with inner or outer ring local faults.

## 2. Bearing Degradation Identification Method

### 2.1. Improved Monopulse Feature Extraction Method

Some researchers have found that the monopulse envelope waveform contains local fault information and can characterize the bearing degradation states. The monopulse feature extraction needs to divide the fault period according to bearing fault characteristic frequency. Its theoretical value is the product of the instantaneous shaft rotation frequency and Fault Characteristic Coefficient (FCC). A different FCC corresponds to different bearing fault types. When the outer ring speed is 0, the FCC calculation formulas are as follows:

(1) $$\;\;\;\;\;\;\mathrm{FCCI}=0.5Z\left(1+\frac{d}{D}\cos\left(\alpha \right)\right),\;\mathrm{FCCO}=0.5Z\left(1-\frac{d}{D}\cos\left(\alpha \right)\right),$$

where FCCI is inner ring FCC, FCCO is outer ring FCC, *d* is the rolling element diameter, *D* is the bearing pitch diameter, and *α* is the contact angle.

**Figure 1:**
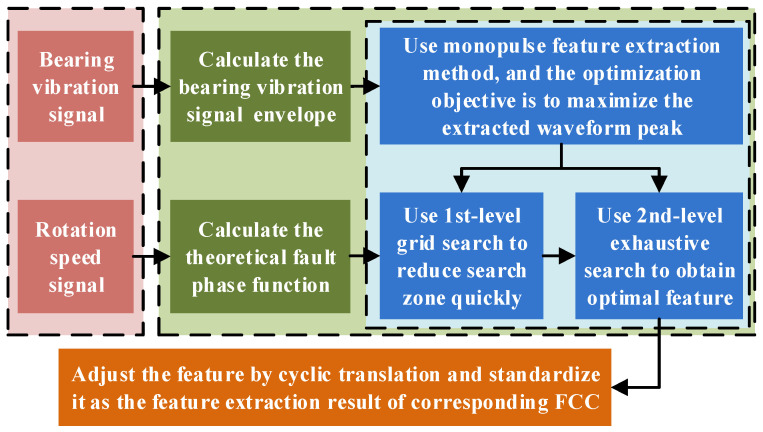
Improved monopulse feature extraction process.

However, due to speed measurement error, FCC calculation error, and relative slip effects, the extracted waveform is prone to smearing. The original monopulse feature extraction method relies on an exhaustive search to calibrate the fault pulse phase, resulting in low computational efficiency. Based on the typical one-dimensional numerical search approach, this paper proposes a two-stage grid search strategy, aiming to significantly reduce the computational load while ensuring accuracy. The improved method process is shown in Figure 1, and its detailed steps are as follows:

Step 1: Calculate the fault phase function *p*(*t*) of the bearing vibration signal *x*(*t*) with duration *T*:

(2) $$p\left(t,\theta \right)=\mathrm{mod}\left(F\int_{0}^{t}f\left(\tau \right)d\tau -\theta ,2\pi \right),$$

where *f*(*τ*) is rotation speed signal, *t* is time, *F* is FCC corresponding to the current fault type, and mod represents the modulo operation. The scanning phase *θ* varies with a step size of 2π/*n* within the scanning range [0, 2π), where *n* is the number of phase scanning steps.

Step 2: Build the key phase function *p*_0_(*t*) based on *p*(*t*):

(3) $$p_{0}\left(t,\theta \right)=\left\{\begin{array}{l} 1,\;\mathrm{diff}\left(p\left(t,\theta \right)\right)<0\\ 0,\;\mathrm{else} \end{array}\right.,$$

where diff denotes the difference operator, and when *p*_0_(*t*) = 1, *t* is the key phase point, which corresponds to the current scanning position.

Step 3: Calculate the average value *P*_0_(*θ*) of the signal envelope at the key phase points as the phase scanning result:

(4) $$P_{0}\left(\theta \right)=\frac{\sum_{t=0}^{T}p_{0}\left(t,\theta \right)h\left(t\right)}{\sum_{t=0}^{T}p_{0}\left(t,\theta \right)},$$

where *h*(*t*) represents the envelope of signal *x*(*t*).

By incrementally increasing *θ*, the average waveform of multiple fault pulses can be calculated, which helps suppress noise interference and compensate for local feature distortion.

Step 4: Calibrate the FCC using a two-stage grid search.

First, set the 1st-level calibration step size *S*_l_ and the step number *N*_1_ to obtain *F_i_* = *F* [1 + *S*_l_ (*i* − *N*_1_ − 1)], *i* = 1, 2, …, and 2*N*_1_ + 1. Therefore, the 1st-level calibration range is ±*S*_l_*N*_1_. Replace *F* with *F_i_*_1_ and repeat Steps 1 to 3 to obtain the corresponding waveform feature *P_i_*. Assume that when *i* = *j*, max(*P_i_*) reaches its maximum value, the global maximum lies within the interval (*F_j_*_−1_, *F_j_*_+1_).

Then, set the 2nd-level calibration step size *S*_2_ and the step number *N*_2_ = (*F_j_*_+1_ − *F_j_*_−1_)/*S*_2_ − 1 to obtain *G_i_* = *F_j_*_−1_ + *FS*_2_*i*, *i* = 1, 2, …, and *N*_2_. Replace *F* with *G_i_* and repeat Steps 1 to 3 to obtain the corresponding waveform feature *P_i._* Assuming that when *i* = *k*, max(*P_i_*) again reaches the maximum value, then *G_k_* is the actual FCC that satisfies the precision requirement. At this point, the waveform feature vector *P*_max_ is selected.

Step 5: Shift the elements in *P*_max_ to align the waveform peak to the center of the feature vector. The missing part at one end is filled by cyclically appending elements from the opposite end. Finally, normalization is performed to scale all data in *P*_max_ to the [0, 1] interval, further mitigating the influence of complex working conditions.

### 2.2. One-Dimensional Dilated Residual Network

**Figure 2:**
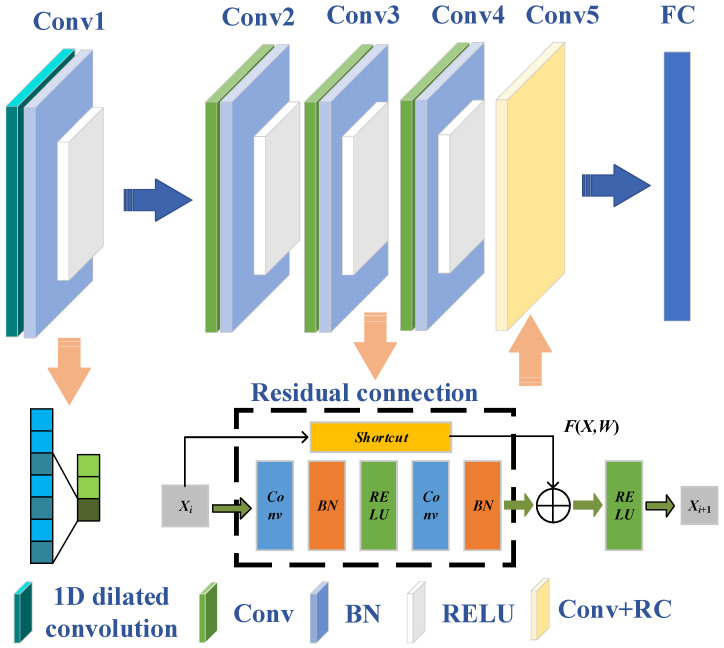
Network model architecture.

In response to the structural characteristics of the extracted feature vectors and the requirements of degradation recognition, 1D-DRCNN is established in this study. The network structure is shown in Figure 2. Compared with conventional convolution, the dilated convolution expands the receptive field without increasing the parameter number by introducing gaps using the dilation factor. The established network takes a one-dimensional single-channel signal of length 1000 as the input. The input data has a time sequence correlation, and the waveform details contain bearing degradation information. Therefore, the larger receptive field helps to reduce feature information loss and improve the overall recognition performance of the model. Initially, a 32-channel 1D dilated convolutional layer is applied. The kernel size is 32 and the dilation rate is 2. Then there are four convolutional layers with decreasing kernel widths of 32, 16, 8, and 8, respectively, while the number of channels varies as 32, 16, 32, and 64 in a decreasing-then-increasing pattern. This design enables the capture of broad local features and facilitates the focus on fine-grained information in deeper layers. Each convolutional layer is composed of a convolution operation, a batch normalization, and a ReLU activation function, enabling efficient multi-level feature integration and improved classification performance. To alleviate the gradient vanishing issue in deeper networks, a skip connection mechanism is introduced. Specifically, the output of the third convolutional layer is transformed by a 1 × 1 convolution and then added to the output of the fifth convolutional layer, forming a residual connection that enhances deep feature fusion. Finally, a fully connected layer maps the extracted features to nine target classes, and the final classification is achieved using a softmax activation followed by a classification layer.

### 2.3. Degradation Identification Method Process

**Figure 3:**
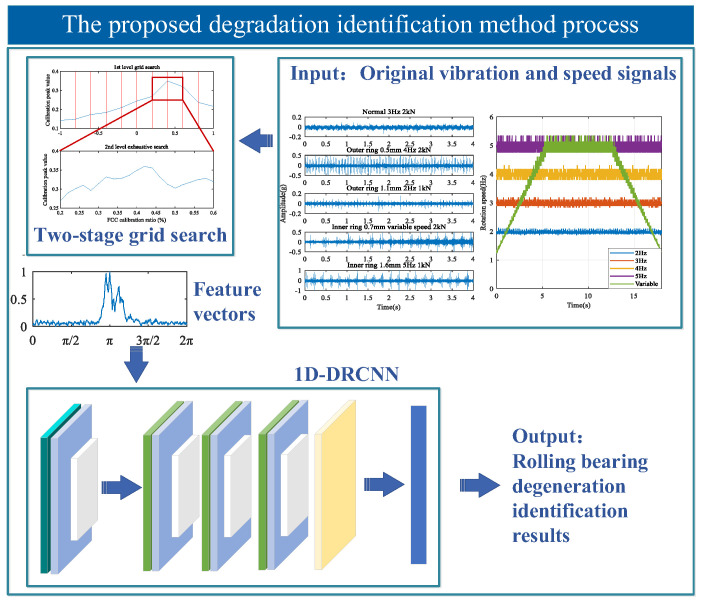
Overall framework of the proposed method.

This study proposes an identification method for rolling bearings under different degradation states, based on an improved monopulse feature extraction and 1D-DRCNN. The method first acquires bearing vibration signals and rotation speed signals, from which the signal envelope and fault phase function can be calculated. Subsequently, the improved monopulse feature extraction approach is employed. The two-stage grid search is used to rapidly calibrate FCC to enhance feature extraction efficiency and mitigate the influence of complex working conditions. This process generates the normalized waveform feature vectors. On this basis, the waveform features extracted using both inner and outer ring FCCs are concatenated to form a comprehensive feature vector. This vector characterizes the overall bearing degradation state. Finally, the 1D-DRCNN is constructed for feature fusion and classification. By making full use of the local features in a monopulse waveform, the model can effectively capture degradation information. Thus, the accurate bearing degradation identification under different working conditions can be achieved. The overall methodological framework is illustrated in Figure 3.

## 3. Data Collection

**Figure 4:**
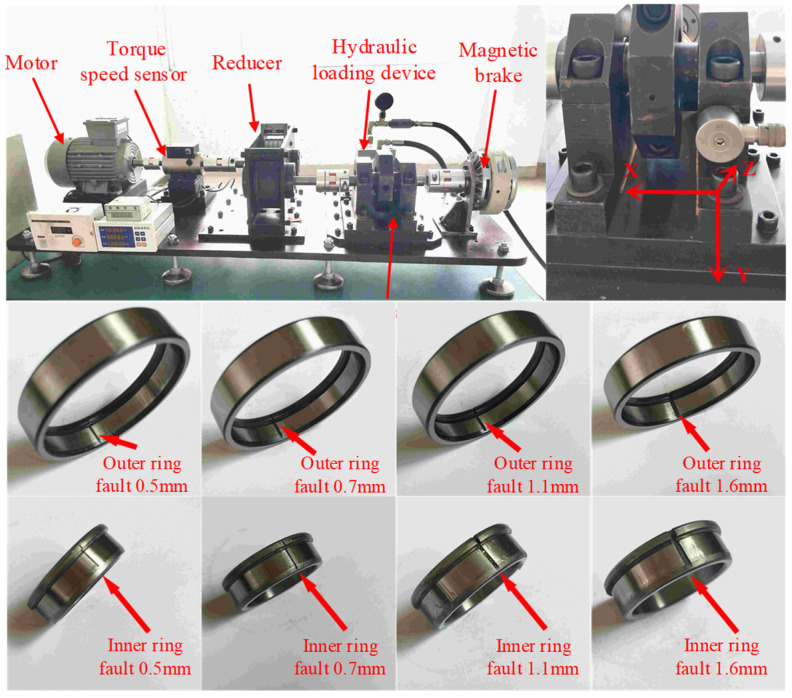
Experimental setup for signal acquisition.

In this study, the NU1007 cylindrical roller bearing was selected as the research object. The outer race diameter is 55 mm, the inner race diameter is 42 mm, the roller diameter is 6.5 mm, and the rolling element number is 16. The experimental setup, sensor placement, and test bearing configuration are illustrated in Figure 4. The radial load was applied using a hydraulic loading device. A triaxial accelerometer was employed to collect the bearing vibration signals, and the sampling frequency was set as 20 kHz. The vibration sensor was mounted on the outer surface of the bearing housing, with the Z-axis signal—perpendicular to the contact surface and directed toward the bearing center—being selected for analysis. Under varying working conditions, the vibration and rotation speed signals of normal bearings and inner and outer race fault bearings with four different fault sizes were collected. All test bearings were installed in the right bearing housing. The four fault sizes are 0.5 mm, 0.7 mm, 1.1 mm, and 1.6 mm, respectively.

## 4. Experimental Analysis

### 4.1. Analysis of Feature Extraction Effect

**Figure 5:**
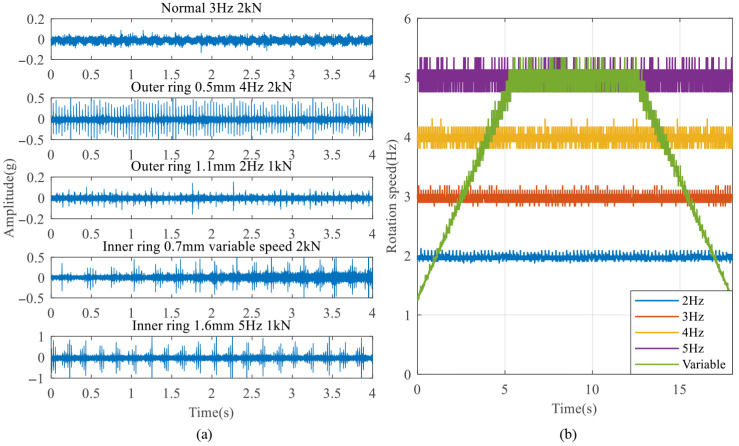
Some collected original data: (**a**) fault bearing vibration signal under different working conditions; (**b**) five rotational speed curves.

The vibration and rotation speed signals were collected for nine different bearing degradation states under five rotation speed conditions and two radial load conditions. The speed conditions include 2 Hz, 3 Hz, 4 Hz, 5 Hz, and a variable-speed condition, while the load conditions include 1 kN and 2 kN radial load. Some acquired data samples are shown in Figure 5a, and five rotational speed curves are shown in Figure 5b. It can be observed that most fault signals exhibit impact components. However, due to the influence of different working conditions, the relationship between impact features and degradation states is complex. In particular, the third sample demonstrates that, although the local defect is relatively large, the overall vibration amplitude appears similar to the normal bearing signal because of the low speed and load. In addition, the normal signal contains a small amount of random weak shocks. The speed curves are also fluctuating and not theoretically continuous and smooth. All of these increase the difficulty of feature extraction and easily lead to misdiagnosis.

**Figure 6:**
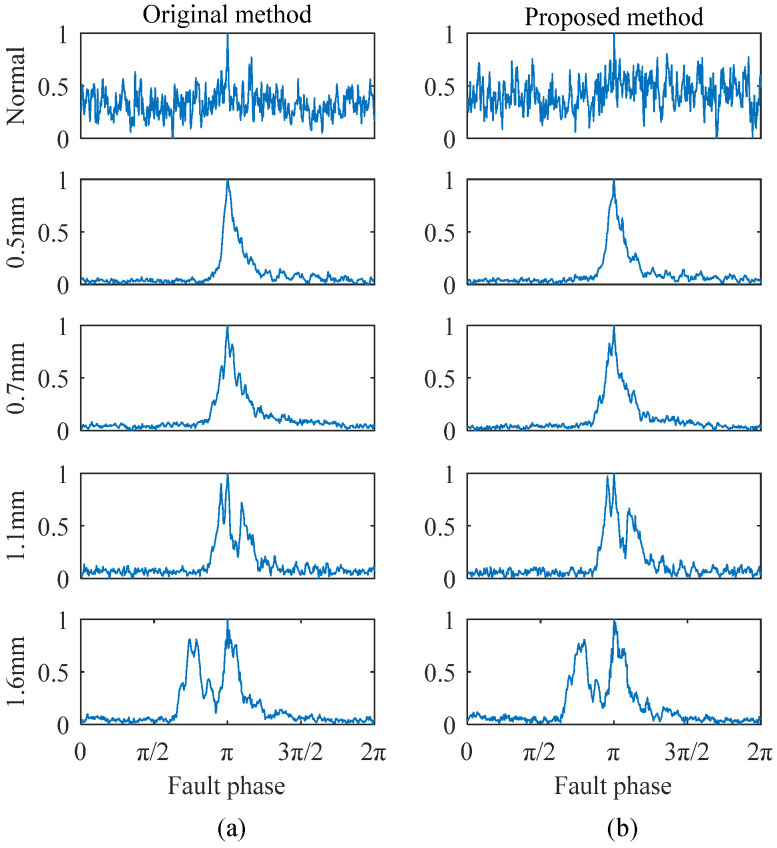
Feature extraction comparison for outer ring faults under 4 Hz and 2 kN conditions: (**a**) original method extraction results; (**b**) improved method extraction results.

**Figure 7:**
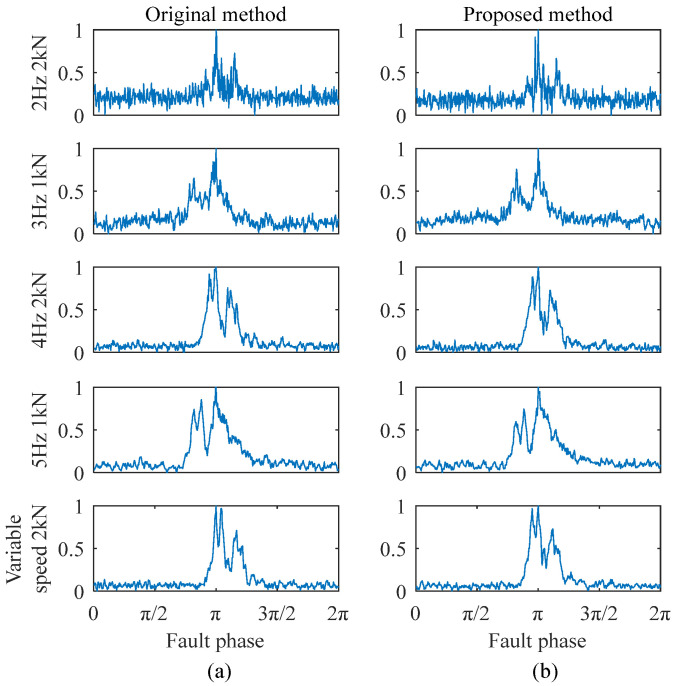
Feature extraction comparison for 1.1 mm outer ring fault under different conditions: (**a**) original method extraction results; (**b**) improved method extraction results.

**Figure 8:**
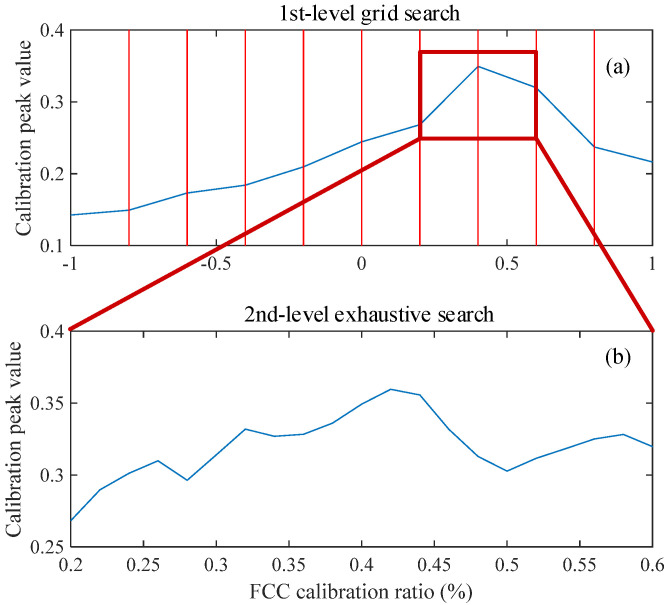
Calibration process of the proposed method: (**a**) 1st-level grid search; (**b**) 2nd-level grid search.

Take the outer ring faults as an example: the proposed improved method and the original method were employed to extract monopulse waveform features, respectively. The original signal samples were segmented over a 1 s time window, and the phase scanning step number was 500. For the original method, the calibration step size was set to 0.02%, with a calibration range of ±1%. For the improved method, a two-stage FCC calibration strategy was employed, with the step size of 0.2% and 0.02%, respectively. This ensured that both methods achieved the same theoretical final calibration precision and maximum calibration range. The result comparisons are shown in Figure 6, Figure 7 and Figure 8.

As shown in Figure 6, the extracted features for different degradation states under the same working condition exhibit significant differences. The peak separation in the monopulse corresponds to the fault size, and there are no obvious double peaks in the monopulse features of a 0.5 mm and 0.7 mm fault. This is because the fault sizes are approximately equal, but there are still some waveform differences that can be seen. As shown in Figure 7, the extracted features of the same degradation state under different working conditions are basically the same, indicating that the working condition influence has been overcome. The results obtained by two methods are generally the same, indicating that both of them can effectively preserve the most direct bearing degradation state information. Therefore, the extraction quality of the improved method can be confirmed.

The key distinction between the two methods is shown in Figure 8. Fundamentally, FCC calibration is a one-dimensional optimization problem aimed at maximizing the peak amplitude of extracted waveform. However, since the objective function is not strictly unimodal within the search interval, applying conventional one-dimensional optimization techniques (such as the golden section method) may easily result in convergence to local optima and lead to calibration failure. The original method adopts an exhaustive search strategy, which guarantees calibration accuracy but comes at the cost of significant computational redundancy. It is because each calibration step requires a full iteration of phase scanning. This leads to inefficiency in feature extraction. Taking the parameter settings in this study as an example, the original method requires 101 iterations. In contrast, the proposed method significantly reduces computational overhead by employing the two-stage grid search strategy. A randomly selected calibration process of the proposed method is displayed in Figure 8. The 1st-level grid search substantially narrows the search interval using 11 search steps, while the 2nd-level exhaustive search ensures calibration precision using 19 search steps. In addition, the midpoint of the 2nd-level search interval can directly use the results from the 1st-level, avoiding redundant computations. With this setup, the proposed method theoretically requires only 29 iterations to achieve the same calibration accuracy. The bearing degradation feature vectors need to be extracted twice using inner and outer ring FCC, respectively. Under the experimental condition using an Intel Core i9-14900K CPU, the total extraction time is approximately 2.3 s. This takes longer than the input original signal and cannot achieve strict continuous real-time diagnosis. However, the bearing performance degradation has a process, and common monitoring strategies are intermittent, such as collecting a 1 s length signal every minute. Thus, the method can meet real-time requirements in practical applications, and less computation also helps to reduce the hardware performance requirements and power consumption of the detection system.

### 4.2. Diagnosis Analysis

**Figure 9:**
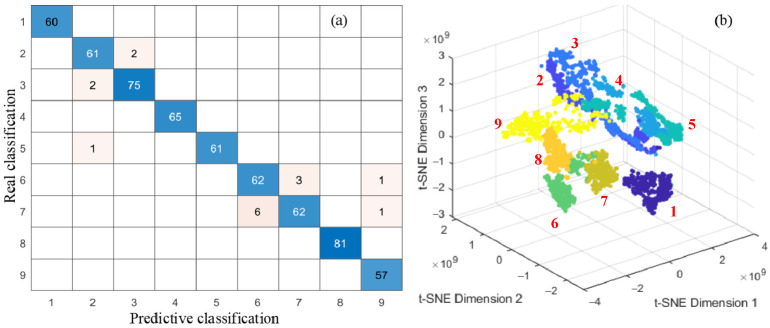
Recognition results and visualization analysis of established model: (**a**) confusion matrix; (**b**) result of t-SNE.

To validate the effectiveness of the proposed method, experiments were conducted using a rolling bearing dataset covering various degradation states. After the feature extraction, a total of 3600 samples were obtained, with each degradation state corresponding to 400 samples. To ensure unbiased sampling, sample indices were randomly shuffled. Among them, 3000 samples were used as the training set and the remaining 600 as the testing set. The network was trained using the Adam optimization algorithm, with a maximum of 30 training epochs and an initial learning rate of 0.001. The L2 regularization (with a weight decay coefficient of 0.0001) was applied to prevent overfitting. The step decay learning rate strategy was adopted and reduced the learning rate by a factor of 0.1 every 400 iterations to promote stable convergence during training. Furthermore, the training data were randomly shuffled at each epoch to improve the model’s generalization ability. The identification results of the proposed method are shown in Figure 9. It should be noted that classes 1–9 represent the normal state and four different sizes of outer ring faults and inner ring faults, respectively.

The experimental results clearly demonstrate the superior performance of the established 1D-DRCNN. The confusion matrix exhibits a well-defined diagonal structure, indicating a high recognition accuracy across most classes and a strong classification capability. Most of the recognition errors are between small-size fault states corresponding to class 2, 3, 6, and 7. Only three cross-category errors confuse large-size faults with small-size faults, and there is no missed diagnosis between healthy and faulty bearing classes. In addition, the deep features extracted from the fully connected (FC) layer were visualized using t-distributed Stochastic Neighbor Embedding (t-SNE), which revealed a clear clustering structure. Samples from different degradation states were well separated in low-dimensional space with distinct class boundaries, which further confirmed the network’s feature discrimination ability and strong intra-class compactness.

### 4.3. Comparative Experiment

To further evaluate and validate the effectiveness of the established model, four representative one-dimensional convolutional neural network (1D-CNN) models were selected for comparative analysis, including DeepConvNet1D, ResNet1D, DilationNet, and InceptionNet1D. All models were tested under identical experimental conditions. The DeepConvNet1D adopts a three-layer sequential convolutional structure, with each layer followed by batch normalization and a ReLU activation function. The ResNet1D incorporates residual connections into the conventional convolutional layers, enabling direct information flow across layers. The DilationNet utilizes dilated convolutions with dilation factors to expand the receptive field without increasing the parameter number. The InceptionNet1D mimics the Inception architecture by employing multi-branch parallel convolutions, with each branch using convolutional kernels of different scales, and the resulting features are concatenated at the channel dimension.

As shown in Table 1, the standard DeepConvNet1D has limited performance with an accuracy of only 91.5%. The InceptionNet1D has achieved an ideal accuracy of 95.17%, indicating that the multi-scale convolutional structure has a good feature extraction ability and can mine most of the fault information. The ResNet1D network with residual structure has improved its nonlinear expression ability with an accuracy of 95.33%. The DilationNet achieved an accuracy of 95.5%, demonstrating that a large receptive field is beneficial for enhancing model performance.

However, although there are structural differences between these models, the performance improvements remain limited. Some high-complexity models exhibit a significant increase in parameter number but without a corresponding gain in accuracy. This suggests that model capacity and recognition performance do not exhibit a strictly linear relationship. In contrast, the established 1D-DRCNN model effectively combines the perceptive ability of 1D dilated convolutions with the deep feature extraction capability of residual structures. This integration enables more comprehensive feature representation, leading to an improved recognition accuracy of 97.33% and validating the effectiveness of the proposed model in rolling bearing degradation identification tasks.

**Figure 10:**
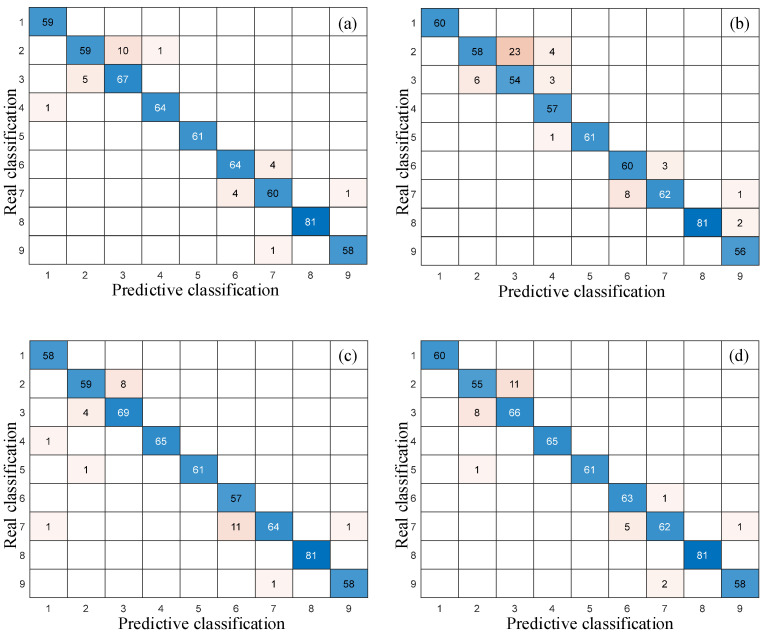
Confusion matrices of comparison models: (**a**) DilationNet; (**b**) DeepConvNet1D; (**c**) ResNet1D; and (**d**) InceptionNet1D.

**Figure 11:**
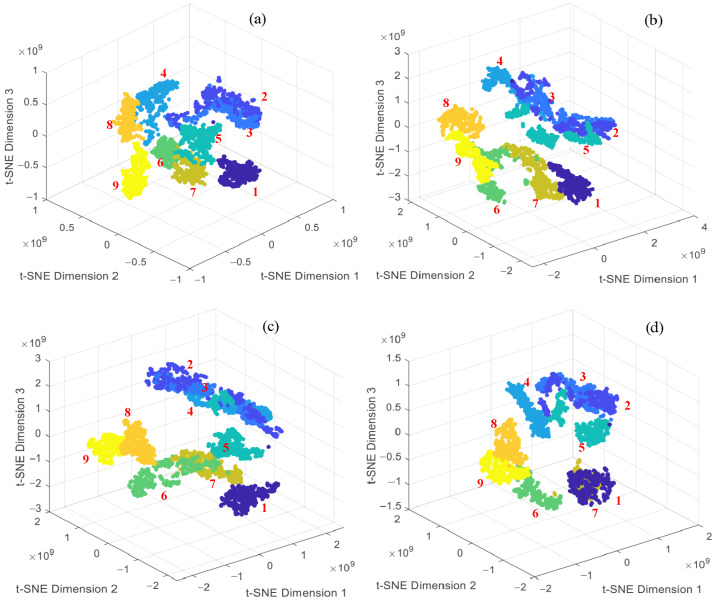
The t-SNE visual analysis of comparison models: (**a**) DilationNet; (**b**) DeepConvNet1D; (**c**) ResNet1D; and (**d**) InceptionNet1D.

Further analysis in Figure 10 and Figure 11 indicates that the performance of other comparison models in multi-classification tasks is relatively poor. These confusion matrices exhibit more non-diagonal distributions, which means more recognition errors. The DeepConvNet1D has 40 recognition errors between small-size faults (0.5 mm and 0.7 mm), and its ability to distinguish similar categories is the worst. The number of small-size fault recognition errors of the DilationNet, ResNet1D, and InceptionNet1D are 23, 23, and 25, respectively, which are still higher than the 1D-DRCNN. Although DilationNet and ResNet1D have good overall recognition rates, they have a one and two confusion between health and fault categories, respectively. This may lead to the missed diagnosis of early faults in practical applications.

Moreover, t-SNE was used to visualize the deep features extracted by these models. The results show that samples from different degradation categories exhibit unclear clustering structures in low-dimensional space, with more inter-class overlap than the proposed model. This is particularly evident in the case of slight damages (e.g., 0.5 mm and 0.7 mm). The class boundaries are blurred, and some samples are intermixed, making it difficult to effectively differentiate between normal and early-stage degradation states. In contrast, the established model demonstrates higher classification accuracy and less class confusion in the confusion matrix. In the t-SNE visualization, samples from different categories form more distinct clusters, indicating stronger feature extraction and multi-class discrimination capabilities. These results validate the robustness and discriminative ability of the proposed method for degradation recognition.

### 4.4. Ablation Study

Finally, ablation studies were conducted to validate the rationality of key parameters of 1D-DRCNN. The influence analysis results of the kernel size and dilation rate on the degradation recognition performance of ID-DRCNN are shown in Table 2. Among all parameter combinations, the structure with the kernel size of 32 and dilation rate of two achieved the best performance. Its accuracy, precision, recall, and F1 score reached 0.9867, 0.9870, 0.9869, and 0.9870, respectively, and are all the highest. When the kernel size is fixed, a dilation rate of two is optimal in most cases. The receptive field is appropriately enlarged while avoiding the problem of excessive sparsity caused by a larger expansion rate. Similarly, when the dilation rate is two, the F1 Scores for the kernel sizes of 8, 16, 32, and 64 are 0.9768, 0.9784, 0.9870, and 0.9700, respectively. Both too small and too large kernel sizes lead to a model performance decrease, and a kernel size of 32 can achieve the optimal balance between the receptive field and feature sensitivity in this task.

Based on the optimal structure mentioned above (KernelSize = 32, Dilation rate = 2), the influence of the input size was analyzed. As shown in Table 3, the recognition results of the 1 × 1000 input structure are better than 2 × 500. One possible explanation is that when the input size is 2 × 500, the feature extraction results of the inner and outer ring FCC will be mixed during the 1D convolution process. This may lead to the mutual interference between unrelated information and recognition performance decrease. In summary, the key parameter selection rationality for the established 1D-DRCNN can be demonstrated.

## 5. Conclusions

(1) The improved monopulse feature extraction method can effectively extract and preserve the normalized waveform characteristics of fault impulses. Compared with the original method, the use of a two-stage grid search strategy significantly reduces the computational cost of FCC iterative calibration while maintaining the same level of calibration accuracy.
(2) For the classification task involving nine similar fault sizes, all tested network architectures achieved accuracy rates exceeding 90%. Among them, the model proposed in this study achieved an overall recognition accuracy of 97.33%. This demonstrates that the monopulse features contain local fault geometric information. It can effectively characterize the bearing degradation states while mitigating the influence of different speeds, loads, and even variable-speed conditions.
(3) The established model employs one-dimensional dilated convolutions to enlarge the receptive field, thereby meeting the requirements for identifying temporally correlated features. The integration of residual connections alleviates issues related to vanishing and exploding gradients, enhancing network learning and generalization capabilities. The comparative analysis of classification, visualization, and ablation study results indicates that the proposed model exhibits better classification performance and is more suitable for bearing degradation diagnosis under complex working conditions.

## Figures and Tables

**Table 1:** Overall identification results of comparison models.

Model	DilationNet	DeepConvNet1D	ResNet1D	InceptionNet1D	1D-DRCNN
Accuracy (%)	95.5	91.5	95.33	95.17	97.33

**Table 2:** Influence analysis of kernel size and dilation rate on established model performance.

Kernel Size	Dilation	Accuracy	Precision	Recall	F1 Score
8	1	0.9767	0.9785	0.9774	0.9780
8	2	0.9767	0.9770	0.9767	0.9768
8	4	0.9800	0.9812	0.9801	0.9806
16	1	0.9800	0.9806	0.9801	0.9804
16	2	0.9783	0.9787	0.9782	0.9784
16	4	0.9783	0.9786	0.9787	0.9787
32	1	0.9850	0.9854	0.9853	0.9853
32	2	0.9867	0.9870	0.9869	0.9870
32	4	0.9717	0.9720	0.9725	0.9723
64	1	0.9725	0.9719	0.9731	0.9725
64	2	0.9700	0.9693	0.9708	0.9700
64	4	0.9658	0.9660	0.9642	0.9651

**Table 3:** Influence analysis of input size on established model performance.

Input Size	Accuracy	Precision	Recall	F1 Score
1 × 1000	0.9817	0.9797	0.9714	0.9706
2 × 500	0.9783	0.9669	0.9657	0.9663

## Data Availability

The data are available on reasonable request from the author.
